# Supplementary material for: Dynamic transcriptomic profiles of zebrafish gills in response to zinc supplementation
Source: BMC Genomics. 2010 Oct 11;11:553. doi: 10.1186/1471-2164-11-553 (PMC3091702; doi:10.1186/1471-2164-11-553)
Supplement: Additional file 2 — Interactive Direct Interaction Network representing the molecular interactions between zinc, copper, iron, calcium and proteins encoded by transcripts changed by zinc supplementation. Mini web-site containing index.html and hyperlinked pages in subdirectory describing a Direct Interaction Network automatically generated based on curated interactions contained within the proprietary PathwayArchitect database. Ovals represent proteins and the circles symbolize metal ions. Objects are coloured by their abundance in zebrafish at the time-point they were significantly different from the control is a scale from -4 fold (dark green) to +4 fold (dark red). Where significant differences were found at more than one time-point, the colour overlay shows expression at the first instance. Dark blue squares denote 'binding', and light blue squares 'expression'; green squares stand for 'regulation', green diamonds for 'metabolism', and green circles for 'promoter binding'. Arrow heads indicate directionality of the interaction where annotated. All nodes and edges can be further interrogated by selecting the relative area of the image. [file 1471-2164-11-553-S2.zip › PathwayArchitect Zn xs DIN/758808.html]

# REGULATION:

|  |  |
| --- | --- |
| Type | REGULATION |
| Effect | None |


---

|  |  |
| --- | --- |
| Score | 0 |


---

|  |  |
| --- | --- |
| Reference Count | 4 |


---

|  |  |
| --- | --- |
| Mechanism | Unknown |


---

|  |  |
| --- | --- |
| Reference:0 || Sentence | "Conversely, excess Ca2+ elicited necrosis and activated calpain but not caspase-3." |
| PMID | 10217261 |
| Year | 1999 |
| Species | Human |
| Journal | J Neurochem |
| RefScore | 1 |
| Source | PArchNLP |
  |
|  |  |


---

|  |  |
| --- | --- |
 Reference:1 || Sentence | "Exocytosis promoted either by a high concentration of PMA or by fMLP is inhibited by leupeptin, indicating that it is due to the action of an intracellular Ca2+-dependent thiol proteinase (calpain), either directly or by conversion by calpain of membrane-bound protein kinase C to the soluble Ca2+/phospholipid-independent form." |
| PMID | 3013847 |
| Year | 1986 |
| Species | Human |
| Journal | J Biol Chem |
| RefScore | 2 |
| Source | PArchNLP |
  ||  |  |


---

|  |  |
| --- | --- |
 Reference:2 || Sentence | "Calpain requires Ca2+ both for proteolysis of its substrates and for interaction with its endogenous inhibitor, calpastatin." |
| PMID | 8034655 |
| Year | 1994 |
| Species | Human |
| Journal | J Biol Chem |
| RefScore | 2 |
| Source | PArchNLP |
  ||  |  |


---

|  |  |
| --- | --- |
 Reference:3 || Sentence | "The significant activation of calpain suggests that calpain activity is regulated not only by an increase in intracellular Ca2+, but also by signaling that include the PKC-, tyrosine kinase- or the adhesion molecule-derived cascade." |
| PMID | 11517927 |
| Year | 2001 |
| Species | Human |
| Journal | Biol Chem |
| RefScore | 3 |
| Source | PArchNLP |
  |


---

|  |  |
| --- | --- |
